# Validation of 1-hour post-thyroidectomy parathyroid hormone level in predicting hypocalcemia

**DOI:** 10.1186/1916-0216-43-5

**Published:** 2014-01-29

**Authors:** Trung N Le, Paul D Kerr, Donna E Sutherland, Pascal Lambert

**Affiliations:** 1 Otolaryngology Head & Neck Surgery, University of Manitoba, Health Sciences Centre, GB421-820 Sherbrook Street, Winnipeg, MB R3A 1R9, Canada; 2 Head and Neck Oncology, University of Manitoba, Health Sciences Centre, GB421-820 Sherbrook Street, Winnipeg, MB R3A 1R9, Canada; 3 Health Outcomes Analyst, Cancer Care Manitoba, ON2114-675 McDermot Avenue, Winnipeg, MB R3E 0 V9, Canada

**Keywords:** Parathyroid hormone, Hypocalcemia, Total thyroidectomy, Completion thyroidectomy, Calcium, Calcitriol, 1-hour PTH

## Abstract

**Background:**

Prior work by our group suggested that a single one hour post-thyroidectomy parathyroid hormone (1 hr PTH) level could accurately stratify patients into high and low risk groups for the development of hypocalcemia. This study looks to validate the safety and efficacy of a protocol based on a 1 hr PTH threshold of 12 pg/ml.

**Study design:**

Retrospective analysis of consecutive cohort treated with standardized protocol.

**Methods:**

One hundred and twenty five consecutive patients underwent total or completion thyroidectomy and their PTH level was drawn 1-hour post operatively. Based on our previous work, patients were stratified into either a low risk group (PTH < 12 pg/ml) or a high risk group (PTH ≥ 12 pg/ml). Patients in the high risk group were immediately started on prophylactic calcium carbonate (5–10 g/d) and calcitriol (0.5-1.0 mcg/d). The outcomes were then reviewed focusing mainly on how many low risk patients developed hypocalcemia (false negative rate), and how many high risk patients failed prophylactic therapy.

**Results:**

Thirty one patients (25%) were stratified as high risk, and 94 (75%) as low risk. Five (16%) of the high risk patients became hypocalcemic despite prophylactic therapy. Two of the low risk group became hypocalcemic, (negative predictive value = 98%). None of the hypocalcemic patients had anything more than mild symptoms.

**Conclusions:**

A single 1-hour post-thyroidectomy PTH level is a very useful way to stratify thyroidectomy patients into high and low risk groups for development of hypocalcemia. Early implementation of oral prophylactic calcium and vitamin D in the high risk patients is a very effective way to prevent serious hypocalcemia. Complex protocols requiring multiple calcium and PTH measurements are not required to guide post-thyroidectomy management.

## Background

Hypocalcemia is one of the most common complications following total thyroidectomy, occurring in 10-50% of cases [1]. It is usually transient, but can be permanent in 0.5–10.6% of patients. It is caused by parathyroid devascularization, stunning, or incidental removal of the parathyroid gland(s) [1-6].

The nadir for hypocalcemia typically occurs at around 24–48 h postoperatively but may be as delayed as post-op day 4 [7]. Therefore, detecting patients requiring calcium replacement therapy with serial calcium measurements can take multiple blood tests over several days. Placing all patients on calcium therapy unnecessarily commits many patients to unnecessary treatment and puts them at risk for hypercalcemia [8]. A clinical laboratory method for early prediction of postoperative hypocalcemia could, therefore, facilitate earlier implementation of treatment, and early discharge (≤24 hours).

In recent years, multiple retrospective and prospective studies have emerged, which support the use of postoperative serum parathyroid hormone (PTH) levels as accurate predictors of hypocalcemia in postoperative thyroidectomy patients. Different groups have published considerable research on this topic, demonstrating that 1 and 6-hour postoperative PTH and Calcium levels had a high sensitivity and specificity of detecting postoperative hypocalcemia [9,10]. Other studies compared PTH percent change as predictor for hypocalcemia [11-13].

Our group previously published a study in which we prospectively followed 42 patients postoperatively with serial PTH and Ca measurements [17]. We found that a that a single postoperative parathyroid hormone (PTH) level, drawn 1 hour after total thyroidectomy, accurately correlated with the development of significant hypocalcemia. All patients who exhibited a PTH level of less than 9 pg/mL 1 hour after surgery developed symptomatic and biochemical hypocalcemia requiring treatment with calcium and vitamin D. None of the patients with PTH ≥ 9 developed hypocalcemia.

Subsequent unpublished data from 100 consecutive total and completion thyroidectomy patients revealed that approximately half of the patients with 1-hour post-thyroidectomy PTH (1 hr PTH) levels in the range of 9–12 pg/ml will develop hypocalcemia. Our center currently categorizes post-thyroidectomy patients as high risk for developing hypocalcemia based on a 1 hr PTH of less than 12 pg/ml. The high risk group is started on prophylactic calcium and vitamin D therapy as soon as the 1 hr PTH level is determined. Those with 1 hr PTH levels of 12 pg/ml or higher are deemed low risk, and are not given prophylactic treatment.

The purpose of this study is to determine the number of high-risk patients who develop hypocalcemia after prophylactic treatment, and to determine the number of low-risk patients who develop hypocalcemia without prophylactic treatment.

## Methods

The study was a retrospective, cohort chart review of consecutive patients undergoing completion or total thyroidectomy from July 2008 to July 2012. All patients underwent a PTH measurement 1 hour post surgery. Based on the result of the 1 hr PTH, patients were divided into a high risk group, (PTH < 12 pg/ml – at high risk for developing hypocalcemia), and a low risk group (PTH ≥ 12 pg/ml – at low risk for developing hypocalcemia).

Patients in the high risk group were started on prophylactic supplementation including calcium carbonate (total dose 5–10 grams per day) and calcitriol (total dose 0.5-1.0 mcg per day) as soon as the PTH level was determined (i.e. within a few hours of surgery). Patients in the low risk group were not given the supplementation.

Both groups were candidates for same day or 23 hour discharge, unless symptoms developed in the interim. All patients were seen in follow-up 1–4 weeks post-operatively. Calcium and PTH levels were rechecked at that time in high risk group to determine the need for ongoing calcium and vitamin D supplementation.

Patient information regarding age, sex, procedure type, final thyroid pathology, and presence of symptoms of hypocalcemia were recorded.

The biologically active, intact 84 amino acid PTH level was measured using the Immulite assay (Diagnostic Products Corporation, Los Angeles, CA), a sensitive and specific two site immunochemiluminometric test. The total incubation time for the Immulite assay is 15–20 minutes, with an analytical sensitivity of 2 to 3 pg/mL. The normal PTH level in our laboratory is 7 to 50 pg/mL. Test results are available shortly after 1 hour from initial sampling. The study was approved by the hospital research ethics committee (Institution Review Board: University of Manitoba Bannatyne Campus Research Ethics Board).

### Surgical technique

Surgical techniques for total and completion thyroidectomy were similar for each operation and all procedures were performed by one of three fellowship-trained head and neck surgeons. Parathyroid glands were preserved whenever possible.

### Overall outcomes

Patients ultimately fell into 4 groups:

1. High risk, eucalcemic (adequate replacement)

These patients had 1 hr PTH level < 12 pg/ml, received calcium and calcitriol supplementation, did not develop symptoms of hypocalcemia, did not have documented biochemical hypocalcemia (corrected calcium ≤1.9 mmol/l), and did not require increased supplementation.

2. High risk, hypocalcemic (inadequate replacement)

These patients had 1 hr PTH level < 12 pg/ml, received calcium and calcitriol supplementation, but developed symptoms of hypocalcemia, had documented biochemical hypocalcemia (corrected calcium ≤1.9 mmol/l), and required increased supplementation.

3. Low risk, eucalcemic (true negative prediction of hypocalcemia)

These patients had 1 hr PTH level ≥ 12 pg/ml, and did not develop symptoms of hypocalcemia, did not have documented biochemical hypocalcemia, and did not require supplementation.

4. Low risk, hypocalcemic (false negative prediction of hypocalcemia)

These patients had 1 hr PTH level ≥ 12 pg/ml, but developed symptoms of hypocalcemia, had documented biochemical hypocalcemia, and required calcium replacement.

### Primary endpoints

We defined two primary endpoints for our study:

1. How many patients do we place at risk by falsely categorizing them as low risk: Negative predictive value of a 1 hr PTH level of ≥ 12 pg/ml?

2. How often do prophylactic oral calcium and vitamin D supplements fail in the high risk group?

## Results

**Figure 1:**
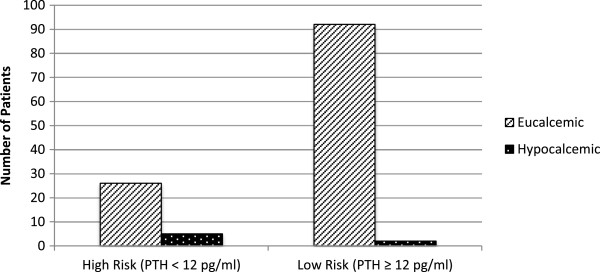
**Proportion of patients in high and low risk groups developing hypocalcemia:**Patients who developed hypocalcemia in the high risk group (5/31) were identified as being likely to develop hypocalcemia, but failed to adequately respond to the initial oral prophylactic calcium and vitamin D supplementation. Patients who developed hypocalcemia in the low risk group are those who were inaccurately categorized by our PTH cut-off (2/94). All the patients who developed hypocalcemia had only mild symptoms. There were no serious sequelae in any patients.

In reviewing of our July 2008 – July 2012 data, there were 125 patients who underwent completion or total thyroidectomy (Figure 1); 94 females and 31 males. One hundred and nine patients underwent total thyroidectomy and 16 underwent completion thyroidectomy. The mean age was 54 years old with a range of 20–87 years. Most procedures were done for oncologic indications. Final histologic diagnoses included 24 multinodular goiters, 3 Grave’s disease, 3 follicular adenomas, and 95 malignancies.

Of the 125 patients, 31 (25%) had a 1 hr PTH level < 12 pg/ml, and were therefore deemed to be at high risk for developing hypocalcemia. Despite this group receiving supplemental calcium and calcitriol, 16% (5/31) developed hypocalcemia (Figure 1). None of these patients developed serious sequelae from hypocalcemia such as tetany or dysrhythmia.

Niney-four patients (75%) had a 1 hr PTH level ≥ 12 pg/ml, and were thus classified as low risk for developing hypocalcemia. Two of these patients (2%) developed hypocalcemia. Thus, the negative predictive value of a 1 hr PTH ≥ 12 pg/ml is 98% (Figure 1). Again, the hypocalcemic patients had only mild symptoms. None of the patients that became hypocalcemic had serious sequelae.

## Discussion

Many authors [9-16] have demonstrated the correlation between post-thyroidectomy PTH levels and the development of hypocalcemia. However, the ensuing management protocols have generally been cumbersome; requiring repeated blood tests and the use of graphs or formulas. Previously published work by our group [17] indicated that a postoperative PTH level < 9 pg/ml, drawn 1 hour after total thyroidectomy, accurately correlated with the development of significant hypocalcemia, leading us to believe that a simpler protocol could be developed. Our unpublished data of 100 subsequent patients between 2003-2007 revealed that approximately half the patients with post-operative PTH levels between 9-12 pg/ml will develop hypocalcemia. Therefore, we have raised the PTH cut-off level from 9 to 12 pg/ml in the development of a protocol for the of a single 1-hour PTH level in predicting the risk of development of hypocalcemia.

Our protocol has an excellent negative predictive value of 98%. It is important to note that the two low risk patients that developed hypocalcemia had borderline PTH levels of 12 and 13 pg/ml. The resulting hypocalcemia did not result in any serious sequelae. One of these two patient became symptomatic within one week of surgery and was started on supplementation. Interestingly, in the second postoperative week they became hypercalemic and the supplementation was stopped. The other developed post-operative hypocalcemia in association with significant hypomagnesemia. This patient also required prolonged hospital stay and intravenous calcium and magnesium supplementation. This case illustrates the potential for other factors such as malnutrition and hypomagnesemia to potentially exacerbate calcium imbalance in these patients. Perhaps magnesium levels should be incorporated into the perioperative management regimen.

While the PTH cut-off could be raised to accommodate these patients, this will inevitably result in a greater number of patients being unnecessarily started on prophylactic treatment. We prefer to leave these borderline patients untreated, but warn them to be extra vigilant about reporting any symptoms so that calcium testing can be promptly performed.

The positive predictive value of our protocol cannot be formally validated with this study as all the high risk patients were given prophylactic treatment. Therefore, it is impossible to determine how many high risk patients would have gone on to develop hypocalcemia. However, we know from our previous series and from literature [1-6] in which high risk patients were not treated that approximately 25% of patients will develop symptoms. This correlates very well with the proportion of patients that our PTH cut-off is identifying as high risk. Therefore, we feel that the positive predictive value is very high, likely in excess of 90%. A high positive predictive value means that very few patients are being unnecessarily placed on supplemental medication or requiring unnecessary follow-up bloodwork.

Our protocol of immediately implementing prophylactic calcium and activated vitamin D supplementation in the high risk group appears to be very effective. From our previous studies, we know that the vast majority of the patients in the high risk group develop symptomatic hypocalcemia if not treated with supplemental calcium and vitamin D. By combining the data of our current study with information from our previous study [17] in which patients from the high risk group were purposefully observed without prophylactic treatment, we found a significant decrease in hypocalcemia among high risk patients receiving prophylactic treatment (OR = 0.05; 95% CI: 0.01- 0.12; p < .001)

While our protocol of prophylactic supplementation failed to prevent hypocalcemia in16% of high risk patients, it is important to note that none of the patients suffered anything more than mild symptoms. There were no serious sequelae.

We have analyzed the 5 cases that were deemed to have failed prophylactic therapy. Factors that appear to be involved in failed supplementation include: compliance, lower doses of calcium and vitamin D, and dietary deficiencies such as hypomagnesemia. All of these patients developed hypocalcemic symptoms within 10 days of surgery. 60% (3/5) of these five patients developed post-operative hypocalcemia associated with documented hypomagnesemia. These patients required prolonged hospital admission or readmission for intravenous calcium and magnesium supplementations. This finding emphasized the importance of magnesium in calcium metabolism. 20% (1/5) of these five patients continue to have long-term hypoparathyroidism (>6 months) and required ongoing calcium and calcitriol supplementations. Some of these patients were strict vegetarians, had low magnesium and/or low vitamin D levels pre-op. This association has been documented in the literature [18].

There was a range in the initial prohylactic supplementation of 5–10 g calcium carbonate and 0.5-1 mcg calcitriol. The five high risk patients that became hypocalcemic were started on the lower end of oral supplementations (5.0 g of calcium and 0.5 mcg of calcitriol per day). Perhaps, we should be routinely dosing between 7.5-10 g/d of calcium carbonate and 0.75-1 mcg/d of calcitriol .

One of the high risk patients developed hypercalcemia on oral supplementation. Therefore, we recommend that a calcium level be drawn 1–2 weeks postop in the high risk group so as to assist in judging the rate at which medication can be tapered. This is echoed by other authors [19].

No predictor of hypoparathyroidism is perfectly accurate. Whatever method of risk categorization is chosen, it is best to consider the result in the full clinical context. How confident is the surgeon regarding the preservation of viable parathyroid glands? What is the preoperative nutritional and biochemical state of the patient? What is the anticipated compliance of the patient? All of these factors can potentially be taken into consideration so as to further lessen risk and improve patient outcomes.

It is important to educate patients regarding to signs and symptoms of hypocalcemia. Our protocol eliminates the need for multiple unnecessary blood tests, or the need for complex formulas and protocols postoperatively. A single 1-hour post-thyroidectomy PTH level of < 12 pg/ml is a very good predictor of hypocalcemia. Complex protocols requiring multiple calcium and PTH measurements are not required to guide post-thyroidectomy management. This simple protocol provides the risk stratification required to safely employ selective calcium and calcitriol supplementation. This facilitates safe, early discharge with informative postoperative instructions for patient and firm follow-up plan.

## Conclusions

A single 1-hour post-thyroidectomy PTH level is a valuable tool for stratifying patients as to their level of risk for developing hypocalcemia. Patients that are deemed high risk should be immediately started on prophylactic supplementation, and can safely be considered for 23-hour discharge. Those deemed low risk can be safely discharged without any supplementation.

## Abbreviations

PTH: Parathyroid hormone; LOS: Length of hospital stay; pg: Picogram; ml: Milliliter; mcg: Microgram; g: Gram; mmol: Millimole; L: Liter; ER: Emergency department; hr: Hour; post-op: Postoperative; cCa: Corrected cacium (based on albumin level); D/C: Discharge home.

## Competing interests

The authors declare that they have no competing interests.

## Authors’ contributions

TL and PK collected and analysed data, and drafted the manuscript. TL and DS applied for research ethics board. PL provided statistical analysis. All authors read and approved the final manuscript.
