# Erratum: Validation of 1-hour post-thyroidectomy parathyroid hormone level in predicting hypocalcemia

**DOI:** 10.1186/s40463-014-0042-6

**Published:** 2014-11-20

**Authors:** Trung N Le, Paul D Kerr, Donna E Sutherland, Pascal Lambert

**Affiliations:** Otolaryngology Head & Neck Surgery, University of Manitoba, Health Sciences Centre, GB421-820 Sherbrook Street, Winnipeg, MB R3A 1R9 Canada; Head and Neck Oncology, University of Manitoba, Health Sciences Centre, GB421-820 Sherbrook Street, Winnipeg, MB R3A 1R9 Canada; Health Outcomes Analyst, Cancer Care Manitoba, ON2114-675 McDermot, Avenue, Winnipeg, MB R3E 0V9 Canada

## Erratum

Following publication of our article [1], it has been brought to our attention that we have incorrectly stated the PTH levels for the low risk and high risk patients in the Methods section in the Abstract.

The correct sentence should be:

- Based on our previous work, patients were stratified into either a low risk group (PTH ≥ 12 pg/ml) or a high risk group (PTH < 12 pg/ml).

We would like to apologise for any confusion caused to our readers.
